# Clinical trials to assess adjuvant therapeutics for severe malaria

**DOI:** 10.1186/s12936-020-03340-3

**Published:** 2020-07-24

**Authors:** Rosauro Varo, Clara Erice, Sydney Johnson, Quique Bassat, Kevin C. Kain

**Affiliations:** 1 ISGlobal, Barcelona Institute for Global Health, Hospital Clínic, Universitat de Barcelona, Barcelona, Spain; 2 Centro de Investigação em Saúde de Manhiça, Manhiça, Mozambique; 3 Sandra-Rotman Centre for Global Health, Toronto General Research Institute, University Health Network-Toronto General Hospital, Toronto, ON Canada; 4 McMaster University, Hamilton, ON Canada; 5 ICREA, Pg. Lluís Companys 23, 08010 Barcelona, Spain; 6 Pediatric Infectious Diseases Unit, Pediatrics Department, Hospital Sant Joan de Déu (University of Barcelona), Barcelona, Spain; 7 Consorcio de Investigación Biomédica en Red de Epidemiología y Salud Pública (CIBERESP), Madrid, Spain; 8 Department of Medicine, Division of Infectious Diseases, Tropical Disease Unit, University of Toronto, Toronto, Canada

**Keywords:** Severe malaria, Angiopoietin-2, Immune and endothelial activation, Microvascular dysfunction, Host-biomarkers, Surrogate endpoints, Drug repurposing

## Abstract

Despite potent anti-malarial treatment, mortality rates associated with severe falciparum malaria remain high. To attempt to improve outcome, several trials have assessed a variety of potential adjunctive therapeutics, however none to date has been shown to be beneficial. This may be due, at least partly, to the therapeutics chosen and clinical trial design used. Here, we highlight three themes that could facilitate the choice and evaluation of putative adjuvant interventions for severe malaria, paving the way for their assessment in randomized controlled trials. Most clinical trials of adjunctive therapeutics to date have been underpowered due to the large number of participants required to reach mortality endpoints, rendering these study designs challenging and expensive to conduct. These limitations may be mitigated by the use of risk-stratification of participants and application of surrogate endpoints. Appropriate surrogate endpoints include direct measures of pathways causally involved in the pathobiology of severe and fatal malaria, including markers of host immune and endothelial activation and microcirculatory dysfunction. We propose using circulating markers of these pathways to identify high-risk participants that would be most likely to benefit from adjunctive therapy, and further by adopting these biomarkers as surrogate endpoints; moreover, choosing interventions that target deleterious host immune responses that directly contribute to microcirculatory dysfunction, multi-organ dysfunction and death; and, finally, prioritizing where possible, drugs that act on these pathways that are already approved by the FDA, or other regulators, for other indications, and are known to be safe in target populations, including children. An emerging understanding of the critical role of the host response in severe malaria pathogenesis may facilitate both clinical trial design and the search of effective adjunctive therapeutics.

## Background

Mortality and morbidity rates associated with falciparum malaria infection remain high. The World Health Organization (WHO) estimated that malaria accounted for 405,000 deaths in 2018 [1], mostly affecting sub-Saharan African (SSA) children [1]. Despite effective treatment with artesunate, between 8.5% and 18% of patients diagnosed with severe malaria (SM) die [2] and up to 50% of cerebral malaria (CM) survivors may develop long-term neurological sequelae [3–5]. The Global Technical Strategy for Malaria 2016–2030 Report calls for at least a 90% reduction in malaria incidence and mortality by 2030 [6]. However, without new and accelerated interventions this goal will not be achieved. Thus, there is an urgent need to develop adjuvant therapies to be used concurrently with anti-malarial drugs to improve clinical outcomes.

SM is a multi-organ syndrome resulting from a complex interaction between both pathogen and host determinants, and its pathophysiology is yet to be fully understood [7]. However, it is becoming increasingly clear that endothelial and immune mediators play key roles in determining disease severity and outcome and thus represent attractive targets for host-directed interventions [8, 9]. There have been multiple efforts to identify adjunctive therapeutics, although to date none of these has been successful [10]. This likely reflects both our limited understanding of malaria physiopathology, as well as the challenges, cost and feasibility of conducting suitably powered randomized controlled trials (RCT) to evaluate mortality outcomes [11]. Most RCTs have relied on specific population sub-groups and were largely underpowered. In addition, study design/characteristics diverge widely between RCTs making it difficult to compare and extrapolate results from the available data [10]. Here, we outline three areas that may help to address limitations of previous efforts to identify effective adjunctive therapeutics.

### Risk-stratification of patients with malaria

In SSA, there are challenges in the early recognition and triage of SM, with as few as 10% of malaria cases appropriately triaged for care and < 30% of SM cases diagnosed and treated promptly, resulting in increased mortality and brain injury in survivors [12, 13]. WHO criteria for SM are commonly used to recruit patients for RCTs [14]. However, these criteria, which are a mixture of clinical and laboratory parameters, are broad, have widely variable prognosis [15], may overlap and can present with other co-morbidities, making it difficult to assess and classify children [16]. Taylor et al. showed, in a post-mortem study, that 23% of children clinically diagnosed with CM, had died from other causes [16]. A recent meta-analysis highlighted the variability between SM-defining criteria and fatal outcomes. Some criteria, such as impaired consciousness, severe anaemia or prostration, are weakly associated, while others, such as renal failure and hyperlactataemia, are strongly correlated with death/outcome [11, 15]. Additionally, the changing epidemiology of SM has caused a shift in its clinical characteristics (e.g., children that develop SM are no longer primarily restricted to < 5 years of age) [17, 18].

It is important to re-evaluate WHO criteria to include emerging insights of SM pathogenesis and new aspects of SM epidemiology. Additionally, complementing WHO criteria with prognostic biomarkers could help identify high-risk patients that would most benefit from RCTs. Histidine-rich protein-2 (HRP-2), lactate, C-reactive protein (CRP) and procalcitonin (PCT), have all shown to be associated with poor outcomes in patients with SM, and have been considered for risk-stratification of children with malaria [19–24]. More recently, host-biomarkers of endothelial and immune activation, which may better reflect the pathological pathways underlying SM, have been identified as independent and quantitative markers of disease severity and outcome in both children and adults with malaria, both in Africa and Asia [25]. The most promising candidates are those that may be involved in casual pathways leading to death such as Angiopoietin-2 (Ang-2), soluble triggering receptor expressed on myeloid cells 1 (sTREM-1), soluble FMS-like tyrosine kinase-1 (sFt-1), soluble tumour necrosis factor receptor 1 (sTNFR-1) and others [26–28]. Additional prospective studies to evaluate their predictive accuracy are required to define their potential clinical utility in triage and risk stratification. The available evidence to date supports Ang-2 as one marker that best addresses the priorities in this article and is also associated with disease severity in *Plasmodium vivax* and *Plasmodium knowlesi* infections [29, 30].

Ang-2, an integral member of the Ang/Tie axis, is a promising candidate for risk stratification and triage. During normal physiological states, the Ang/Tie axis is involved in maintaining endothelial integrity through the binding of Angiopoietin-1 (Ang-1) to its receptor Tie-2. SM triggers a pro-inflammatory environment which promotes the expression and release of Ang-2, the antagonist of Ang-1, which competes for binding to Tie-2 and destabilizes the microvasculature [31]. Preclinical studies in mice have shown a casual and mechanistic link of the Ang/Tie axis in the pathogenesis of SM [32]. Data from human studies strongly support Ang-2 as an excellent biomarker for malaria disease severity and related multi-organ dysfunction and death; consequently, Ang-2 is a valuable new option for identifying high-risk patients for RCTs [26, 27, 33–35]. Ang-2 plasma concentrations are higher in children with SM compared to those with uncomplicated malaria (UM) [27, 34, 36, 37], and have also been linked to CM with retinopathy [36]. Importantly, the identification of retinal changes in children with CM has been a major advance in the risk-stratification of those patients [38].

### Searching for surrogate endpoints of mortality

Conducting RCTs can be costly and time-consuming and in low-and middle-income countries the challenges are even greater [11]. To demonstrate efficacy of adjunctive therapeutics in reducing mortality requires the enrolment of very large numbers of participants, which may be untenable due to cost and/or logistics. Power calculations indicate that at least 30,000 participants would have to be enrolled in order to observe a 10% change (parting from a 9% mortality rate) [11]. In an effort to address this problem the Severe Malaria African Children: A Clinical Network (SMAC) was created [39]. This was a multicentre pan-African effort to coordinate RCTs with mortality endpoints. Still, with such a network in place, it may take 3–4 years to enrol the required participants, meaning only a limited number of interventions can be assessed [11, 39]. Ultimately, underpowered studies can result in the inappropriate rejection of novel therapeutics because of their failure to show beneficial effects [11]. The identification of new surrogate endpoints, such as biomarker levels, might help address these problems. However, it is important to note that mortality should always be measured as a secondary endpoint in these RCTs, to allow a better characterization of the trends and relationships between levels of biomarkers and groups of treatment.

An appropriate surrogate endpoint should be able to predict/measure a clinical outcome for a specific intervention and be part of the casual pathway of the disease. This is particularly true when considering biomarkers, as if they are not direct readouts of the underlying pathobiology of SM, but rather just correlated to disease outcome, they may lead to confounding findings. Moreover, biomarkers used as surrogate endpoints and the intervention being assessed should also converge on the same pathways [40]. To date, the only proposed surrogate endpoint that has been validated for SM is plasma lactate. A secondary analysis, on three datasets from clinical studies looking at anti-malarial efficacies, showed that measuring changes in plasma lactate concentration at 8 or 12 h after intervention is a valid surrogate endpoint for mortality for treatments aiming to improve microcirculation [11]. However, lactate has a number of limitations discussed in detail by Jeeyapant et al. [11]. Briefly, these include that only a proportion of patients with SM will present with metabolic acidosis and that patients have poor outcomes related to multiple organ dysfunction (e.g., coma or acute kidney injury). Therefore, adjunctive therapies could improve survival through mechanisms that do not involve lactate clearance, and interventions that reduce lactate may not be effective adjunctive therapies.

In contrast to lactate, the Ang/Tie2 axis has been shown to have a causal relationship to severity and death for malaria [32] and Ang-2 concentrations are associated with multi-organ dysfunction leading to death, including acute kidney injury and coma [26, 41]. High Ang-2 concentrations have been linked to multi-organ dysfunction and mortality for multiple causes of sepsis, including malaria [27, 42–45]. Specifically, Ang-2 has been demonstrated to be elevated in patients with SM and to be an independent and quantitative predictor of mortality [27, 33]. Importantly, Ang-2 levels at admission are higher in children who die in hospital, as well as being associated with longer recovery times in survivors and post-discharge mortality [26]. Reduction in plasma levels of Ang-2 has already been used as a primary outcome in a RCT assessing inhaled nitric oxide as adjunctive therapy for paediatric SM [46]. Moreover, interventions targeting this pathway improve outcome in preclinical models [32, 47]. Taking into consideration the central role that endothelial activation and microcirculatory dysfunction play in SM pathogenesis and the mechanistic link that the Ang/Tie axis plays, we propose Ang-2 as another possible surrogate endpoint candidate, either alone or in conjunction with other markers such as lactate. Furthermore, lactate can already be measured using a point-of-care (POC) test and there is ongoing research trying to design similar POC devices for Ang-2 and other markers. This could facilitate the implementation and impact of marker-based risk-stratification in resource-constrained settings.

### Drug repurposing

Identification of novel therapeutics is expensive, time consuming and risky, with many promising new chemical entities never reaching or showing efficacy in Phase III trials. In the field of cancer research, it has been estimated that de novo therapeutic development takes between 10 and 17 years with cost estimates of 1–2 billion USD [8]. However, this can be de-risked, at least in part, by drug repurposing, which involves the search of new therapeutic indications for already marketed drugs with known safety profiles [48]. With this strategy, success rates may be enhanced with dramatically reduced costs and timelines to RCTs [8, 49, 50]. Therefore, drug repurposing is an attractive avenue for therapeutic development in common and rare diseases, including SM [8, 49, 50].

The primary hurdle in drug repurposing is the identification of appropriate drugs to test. A multitude of databases, data mining tools and compound libraries are emerging to help the scientific community sift through the plethora of potential candidates [50]. For example, Repurposing, Focused Rescue, and Accelerated Medchem (ReFRAME), is an open access screening library of 12,000 compounds compiled from commercial drug competitive intelligence databases [51]. Such tools could be used towards identifying adjunctive therapeutics for SM that target either deleterious host immune responses and/or protect/stabilize the microvasculature. A recent review explores the advantages and challenges of using licensed pharmaceuticals, developed originally as therapy for cancer and neurological disease, as possible candidates for CM. Furthermore, they emphasize the importance of targeting pathways of microvascular stability and blood brain barrier (BBB) function [52]. However, an accelerated strategy will still require that any promising candidate be prospectively evaluated in phase II RCTs and then, if proven to be effective, further assessed in larger Phase III trials evaluating adverse events and mortality before they can be more widely implemented.

A direct example of drug repurposing used in the context of SM is rosiglitazone [53, 54]. Rosiglitazone, a peroxisome proliferator-activated receptor (PPARγ) agonist, with immunomodulatory activity and capacity to promote endothelial integrity, was originally developed to treat type II diabetes. PPARγ-agonists were initially investigated because they were predicted to act on similar gene response elements as vitamin A metabolites (e.g., 9-cis retinoic acid), which were associated with protection in malaria preclinical models and in vitamin A malaria studies [55, 56]. Current evidence supports its utility to modulate multiple pathways in malaria pathogenesis. Preclinical models have shown that rosiglitazone reduces levels of Ang-2, increases levels of Ang-1, stabilizes the BBB and is neuroprotective [47, 57]. Adjunctive treatment with rosiglitazone has been shown to decrease inflammatory biomarkers associated with adverse outcomes, and reduce parasite burdens in adults [54]. In addition, rosiglitazone has been demonstrated to be safe and well tolerated in children with UM [53]. Cumulatively, this has led to its assessment as an adjuvant therapy in children with SM in an ongoing Phase II clinical trial (clinicaltrials.gov: NCT02694874). The primary endpoint of which is to determine whether rosiglitazone, in addition to parenteral artesunate (standard of care anti-malarial treatment), accelerates the rate of decline in Ang-2 from admission levels, compared to standard of care plus placebo. Atorvastatin is another FDA-approved drug that has been suggested as a possible adjuvant therapy due to its anti-inflammatory and neuroprotective effects [9].

### Current barriers for biomarker implementation

The future use of Ang-2 and other biomarkers in RCTs has some important limitations that need to be considered. Although these molecules are independent and quantitative markers of severity and outcome, it is unlikely that any single clinical or laboratory measurement will be uniformly predictive. Therefore, algorithms that combine predictive clinical (e.g., LODs [58] or qSOFA [59]) and marker data may ultimately be most predictive. Importantly, these algorithms still need to be developed and validated. Moreover, evaluation of baseline malaria mortality (irrespective of being recruited to a trial using biomarkers for risk-stratification) in the study population will need to be conducted, and would allow a better understanding of ‘real mortality risk’ in those not captured by biomarker levels. In addition, there is a clear variability in the thresholds/cut-offs and confidence intervals (CI) currently reported for biomarkers (including lactate and Ang-2) in association with mortality endpoints. There are many technical and methodological issues that may contribute to this variability and that currently preclude providing specific data on cut-offs/ranges. These include: the sample source (finger-prick *versus* venipuncture) and matrix used (whole blood, plasma (EDTA, heparin, etc.), serum); fresh *versus* frozen samples; the platform used to detect and quantitate the marker(s) (e.g., ELISA, Luminex™, ELLA™, etc.); patient population (adult, paediatric, underlying disease, HIV-1 infection).

What is clear is that there is an urgent need for rigorous prospective evaluation of candidate markers head-to-head under standardized protocols to first determine, and then validate cut-offs and CIs in further multi-site prospective studies. These studies have not yet been rigorously conducted and these issues will remain major barriers to the use of surrogate markers as endpoints of studies.

## Conclusions

**Fig. 1:**
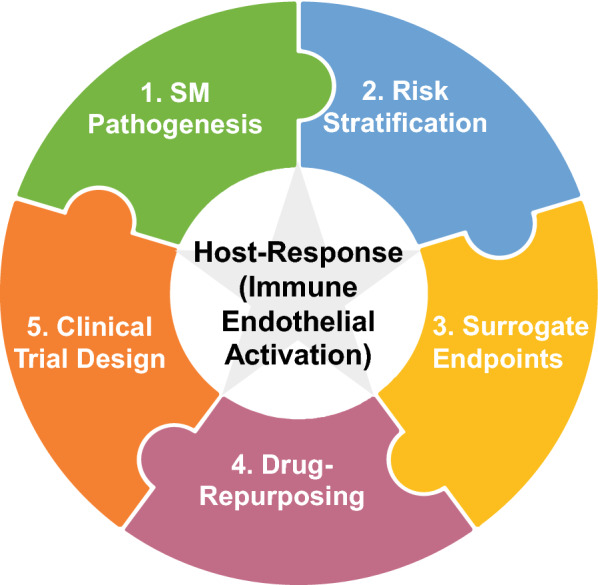
Dysregulated host immune and endothelial activation as the rationale to enhance clinical trial design and identify adjunctive therapeutics for severe malaria. The host-response plays a central role in the pathogenesis and outcome of severe malaria (SM). Therefore, measuring levels of biomarkers of immune and endothelial activation, could be used both to identify patients that would benefit most from randomized control trials and as surrogate endpoints. FDA-approved drugs that protect and/or stabilize the host microvasculature and/or that are immunomodulatory could be repurposed as adjunctive therapeutics for severe malaria. These candidate therapeutics should be paired with the enhanced design of clinical trials

Our improved understanding of the pathobiology of SM should facilitate enhanced clinical trial design. Specifically: by decreasing required sample sizes by using biomarkers (e.g., Ang-2) to risk-stratify children and adults into RCTs; through the use of validated surrogate endpoints of mortality; and, via the search for safe FDA-approved drugs that modulate these underlying causal pathways (Fig. 1).

## Data Availability

Not applicable.

## Abbreviations

Ang: Angiopoietin
BBB: Blood brain barrier
CI: Confidence intervals
CM: Cerebral malaria
CRP: C-reactive protein
HRP-2: Histidine-rich protein-2
PCT: Procalcitonin
PPARγ: Peroxisome proliferator-activated receptor
POC: Point-of-care
RCT: Randomized controlled trial
SM: Severe malaria
SSA: Sub-Saharan Africa
UM: Uncomplicated malaria
WHO: World Health Organization
